# Negative Capacitance in Nanocomposite Based on High-Density Polyethylene (HDPE) with Multiwalled Carbon Nanotubes (CNTs)

**DOI:** 10.3390/ma16144901

**Published:** 2023-07-09

**Authors:** Raymonde Mouecoucou, Leïla Bonnaud, Philippe Dubois

**Affiliations:** 1 Laboratoire Matériaux Optiques, Photonique et Systèmes (LMOPS), Université de Lorraine, 2 Rue Edouard Belin, 57070 Metz, France; ray.mouecoucou@yahoo.fr; 2 Laboratory of Polymeric and Composite Materials (LPCM), Center of Innovation and Research in Materials, Materia Nova Research Center & University of Mons (UMONS), Place du Parc 20, 7000 Mons, Belgium; philippe.dubois@umons.ac.be

**Keywords:** negative capacitance, dielectric organic material, nanocomposites, polyethylene, carbon nanotubes

## Abstract

Negative capacitance (NC), already observed in conducting polymer-based nanocomposites, was recently reported and evidenced at low frequencies (<10 kHz) in non-conducting polymer-based nanocomposites containing conductive particles. In this contribution, we demonstrate that it is possible to produce economic high-density polyethylene (HDPE) nanocomposites exhibiting an NC effect at low frequencies via a convenient and environmentally friendly extrusion-like process by only adjusting the duration of melt-mixing. Nanocomposite materials are produced by confining a limited quantity, i.e., 4.6 wt.%, of multiwalled carbon nanotubes (CNTs) within semi-crystalline HDPE to reach the percolation threshold. With increasing melt processing time, crystallites of HDPE developing at the surface of CNTs become bigger and perturbate the connections between CNTs leading to a dramatic change in the electrical behavior of the systems. More specifically, the link between NC and current oscillations is stressed while the dependence of NC with the size of polymer crystallites is evidenced. NC tends to appear when space charge effects take place in HDPE/MWCNT interfaces, in structures with convenient crystallite sizes corresponding to 10 min of melt-mixing.

## 1. Introduction

Negative capacitance (NC) in electronics expresses the fact that a capacitive device behaves like an inductor one.

NC is a key property much sought-after in electronics, as its application potential is very broad in fields such as electromagnetic absorption, wireless power transfer, field effect transistors, actuators, sensors, supercapacitors and energy harvesting, among others [1,2].

NC research generally concerns two types of devices and materials. The first type is based on semi-conductor devices and materials with precise periodic geometries [3,4,5,6,7,8,9,10,11,12]. Their main drawback is precisely that NC originates from their artificial periodic configuration and their size depends on the wavelength of the applied electromagnetic field, making it very difficult to change the shape or miniaturization and limiting their scalable manufacturing [1,2,3,4,5,6,7,8,9,10,11,12]. The advent of nanotechnologies has opened up a whole new field of possibilities for a second type of material—i.e., nanocomposites—making it easier and more robust to fabricate randomly ordered multi-phase materials by dispersing and confining electroconductive nanoparticles in a matrix that is not necessarily electroconductive. One of the main advantages of such systems is their ability to exhibit both positive and negative values of capacity by tuning their percolation threshold (i.e., critical composition from which the formation of a 3D electroconductive network is obtained with the dispersed phase and free charge carrier conduction takes place) [1].

Interestingly, this nanocomposite approach has been focused largely on the study of systems based on ferroelectric or metallic nanoparticles embedded in rigid ceramic matrices of high dielectric permittivity [13,14,15,16,17,18,19] or in a few generally semiconducting polymers [20,21,22,23,24,25]. Relatively recently, more and more studies have begun to appear on the dispersion of carbonaceous electrically conductive nanofillers such as carbon nanotubes, nanofibers or graphene sheets in polymeric systems to fabricate systems with NC properties [1,2,26,27,28,29,30].

Among the relevant developments, Song et al. [26] recently prepared polyvinylidene fluoride (PVDF)/carbon nanotube (CNT) nanocomposites with different filler content by hot-pressing. They chose PVDF for being an excellent dielectric and because its crystalline beta phase is known to have ferroelectric and piezoelectric properties. They observed that the NC of the composites changed from positive to negative from and above the percolation threshold (between 8 and 10 wt.%), and with the further increase in CNT content, they observed an increase in the absolute value of NC (permittivity from −3000 down to −9000 at 20 Hz for 10 wt.% and 14 wt.% of CNTs, respectively).

Swetha et al. [1] also observed the capacitance to inductive transition as a result of electrical percolation during filler loading (1.25 wt.%) in the nanocomposites, which they produced with polyvinyl alcohol (PVA) and carbon black (CB) particles by die-casting. By further increasing CB content to 2.5 wt.%, they achieved higher NC (permittivity −443 at 15 kHz).

A similar trend was observed by Wu et al. [27] with acrylic polyurethane/graphene nanocomposites. Strangely, although they reported a percolation threshold at 1.8 Vol% of graphene, they seemed to observe NC at 3 Vol%—unfortunately, no explanation was given.

Li et al. [28] were among the first ones to show that metallic particles are not indispensable to prepare nanocomposite materials exhibiting NC. They elaborated polyimide (PI)/carbon nanofibers (CNF) and polyetherimide (PEI)/CNF nanocomposite films obtained by solvent casting. In their study, they used different types of CNF (herringbone CNF and cupstacked CNF) and also modified the length of the cupstacked CNF by ultrasonic cutting. They showed that NC levels were dependent on the filler loading, the aspect ratio and microstructure of the nanofibers, as well as the chemical structures of the polymer matrix. More precisely, they attributed the higher NC of PI/CNF (permittivity of −225 at 5 kHz for 2 wt.% CNF) compared with PEI/CNF (permittivity of −20 at 5 kHz for 2 wt.% CNF) to the higher polarity of imide groups compared to ether groups, which promote the interfacial interactions between the polyimide chains and nanofillers, and thus, the interfacial polarization.

The effect of the structure of the carbonaceous nanofiller on NC was studied by Wu et al. [29] in phenolic-based nanocomposites. More precisely, they prepared phenolic resin (PR) nanofilled with different content of graphene (GR) from 1.4 to 12 wt.%. They achieved NC at low frequencies only when GR content reached 12 wt.%. When they substituted part of the GR with an equivalent amount of carbon nanotubes (CNTs), they found it was possible to improve the NC level for appropriate replacement amounts of CNTs (3 wt.% and 6 wt.%). Moreover, they were also able to obtain nanocomposites exhibiting NC at much lower total carbon content than 12 wt.% by combining 1.6 wt.% of CNTs with 5 wt.% of GR. The high aspect ratio of CNTs was found to be beneficial to the formation of continuous conductive pathways by promoting connections between GR nanosheets.

Zhu et al. [30] synthesized electroconductive nanocomposites based on graphene of different sizes, carbon nanotubes (CNTs) and carbon nanofibers (CNF) integrated with semiconductive polypyrrole (PPy) by surface-initiated polymerization. They studied the effect of different filler ratios (from 0.1 to 5 wt.%). In their case, as a result of the semiconductive nature of the PPy matrix, all the nanocomposites were found to exhibit NC and to be dependent on the filler loading, the morphology and surface functionality of the particles.

Finally, and very recently, Wang et al. [2] observed NC in flexible thermoplastic polyurethane (TPU)/carbon nanotube (CNT) nanocomposites with quite a high amount of CNTs (>20 wt.%).

Surprisingly, to our knowledge, no study has yet looked at the use of a traditional commodity polymer such as polyethylene (PE). PE is one of the world’s most widely used thermoplastics. It offers excellent resistance to impact, pressure, abrasion and temperature variations. It is also highly resistant to chemical agents (acids, greases or hydrocarbons). It is easily processed to give it the desired appearance by melt-blending processes such as extrusion, which are environmentally friendly compared to solution processes.

To reinforce and make it conductive, carbonaceous nanofillers can be added. This is a subject that has been extensively addressed by both academia and industry [31,32,33,34,35,36,37]; today, these CNT-based polymer nanocomposites find applications as electrostatic discharge (EDS) coatings [35] and electromagnetic interference (EMI) shielding [36,37].

In this contribution, we look at the possibility of extending the uses of HDPE to the field of electronics. More specifically, we focus on the detection of possible NC behavior in HDPE/CNT nanocomposites for a composition of 4.6 wt.%. This CNT content was chosen in particular because it corresponds to the percolation threshold of the HDPE/CNT system at which the CNTs form an interconnected network enabling the delocalization and free transport of electrons leading to the appearance of particular properties such as the NC effect. The amount of 4.6 wt.% was established in previous work on this type of nanocomposite and was reported in a previous publication [32].

## 2. Materials and Methods

### 2.1. Materials and Samples Preparation Conditions

The carbon nanotubes (CNTs) used in this study were multiwalled carbon nanotubes provided by Future Carbon GmbH. They were thin homogeneous tubes with 10–20 concentrically bent single graphene layers with an average diameter of ~20 nm and purity of >98%. The matrix of the study was a high-viscosity Ziegler Natta HDPE (grade MS201 BN-NA from Total Petrochemicals—see Table 1).

An internal mixer (Brabender, Duisburg, Germany) was used to prepare all composites following a masterbatch dilution approach, which is a common method used in industrial applications. First, a masterbatch (MB) with CNT loading of 20 wt.% was produced at 190 °C in the melt by 3 min mixing at 30 rpm (for introduction of materials) followed by 2 min mixing at 60 rpm. The resulting masterbatch was then diluted at 190 °C, 3 min at 30 rpm and further for either 2 min at 60 rpm for samples 1 or 7 min at 60 rpm for samples 2. For further characterization, the samples were compression molded after extrusion. The same protocol was followed for each sample. An AGILA PE ZO Hot Press was used to mold the samples. The experimental details can be found in reference [32]. Nanocomposites hence produced display crystallite/interface structures [32].

### 2.2. Characterization of Materials

The mechanical, thermal and electrical properties of the produced composites were evaluated.

For mechanical properties, tensile tests and impact strength were determined. More precisely, hot-pressed samples in dog-bone shape were tested in a tensile test instrument LLOYD LR10K (Lloyd Instruments Ltd., Bognor Regis, West Sussex, UK) at a speed rate of 10 mm/min using a distance of 58 mm between grips. Each kind of sample is prepared into 5 specimens previously conditioned for at least 48 h at 20 ± 2 °C under a relative humidity of 50 ± 5% in order to ensure statistical reliability. The tensile test was realized following ASTM D638 type V. Regarding the impact strength test, 5 notched bars (1 × 6 × 0.3 cm^3^) of each sample were prepared with a Ray-Ran 1900 notching apparatus (Ray-Ran Test Equipment Ltd., Warwickshire, UK) and conditioned for at least 48 h at 20 ± 2 °C under a relative humidity of 50 ± 5% to release the constraints. Then, the specimens were tested following standard ASTM D256 using a Ray-Ran 2500 pendulum impact tester.

For thermal properties, differential scanning calorimetry (DSC) measurements were performed to study the non-isothermal crystallization of the produced samples by using a DSC 2920 from TA Instruments calibrated with indium under nitrogen flow (50 mL/min). Samples of about 10 mg were first heated from room temperature to 190 °C (1st heating) at 10 °C/min and kept at this temperature for 5 min. Next, the samples were cooled down to room temperature at a constant cooling rate of 10 °C/min and, finally, heated back to 190 °C (2nd heating). The thermal parameters (i.e., melting temperature (T_m_) and melting enthalpy (ΔH_m_)) were determined from the first scan, which was representative of the thermal history of the samples. More specifically, the melting enthalpy (ΔH_m_) of the samples was used to determine the degree of crystallinity (DC) of the samples following the classical equation: DC = ΔH_m_/ΔH0_m_ × 100, where ΔH0_m_ is the melting enthalpy of 100% crystalline HDPE considered 293 J/g. All data were normalized to the amounts of HDPE from the samples.

For electrical properties, DC resistivity and AC dielectric measurements were performed. More specifically, for DC electrical measurements, specimens having a disk shape (10 cm diameter and 1 mm thickness) were prepared. An electrometer (6517B Keithley) combined with a 8009 BOX from Keithley were used to perform the measurements at 100 V and 25 °C. For each sample, three specimens were tested. For dielectric measurements, the specimens used have dimensions 3 cm × 1.2 cm × 0.3 cm and a 200 nm thick gold electrode entirely coated both faces of the HDPE-CNT nanocomposite samples. Those electrodes were deposited on each face by the method of vacuum evaporation. Those deposits were made by effect joule evaporative with EDWARDS Auto306A, and the crucibles used were of the type ME4-005MO. Frequency dependence of capacitance C and of conductance G were measured using a Keithley 4200-ScS impedance analyzer in parallel mode configuration under ac voltage 0.1 V in the range of 5–50 kHz for different applied bias voltages varying from 0.5 to 4.5 V to obtain very precise capacitance measurements. The real permittivity was calculated by $\varepsilon_{r}=\frac{tC_{p}}{A\varepsilon_{0}}$, where “t” is the thickness of the sample, “A” is the area of the electrode and “$\varepsilon_{0}$” is the absolute permittivity of free space (8.85 × $10^{-12}\;F/m$). For the study of the trend, we consider $\frac{t}{A}=1$ and, for clarity, fitting will only be represented for one curve, for each sample.

## 3. Results and Discussion

### 3.1. Preliminaries Characterizations of the Composites

Nanocomposites based on HDPE matrix with CNT concentration of 4.6 wt.% were prepared and characterized in terms of thermal, mechanical and electrical properties. The results are gathered in the Table 2.

**Figure 1:**
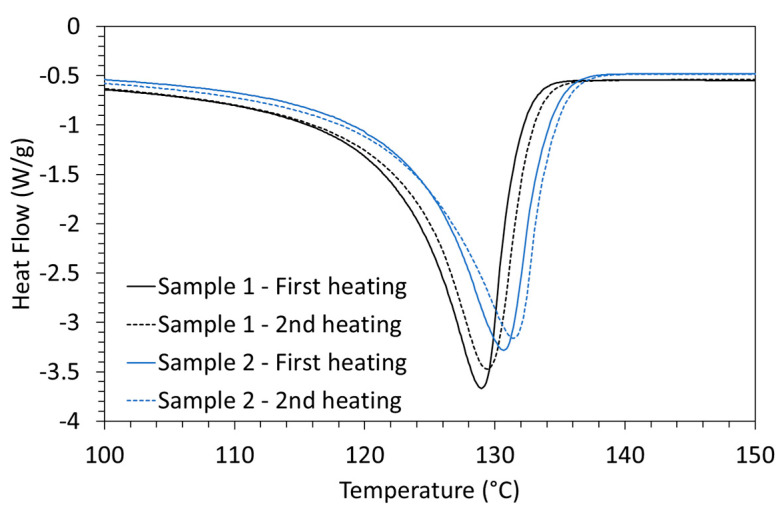
DSC heating curves for HDPE/4.6%CNT nanocomposites (as prepared by melt blending with processing times of 10 min and 20 min) at a heating rate of 10 °C/min.

Regarding the thermal properties, as obtained from the first scan of thermal heating, a single melting peak is observed by DSC for all the nanocomposites (Figure 1). The resulting melting enthalpy and calculated degree of crystallinity are similar for all the nanocomposites (respectively, about 175 J/g for the melting enthalpy and about 60% for the degree of crystallinity).

**Figure 2:**
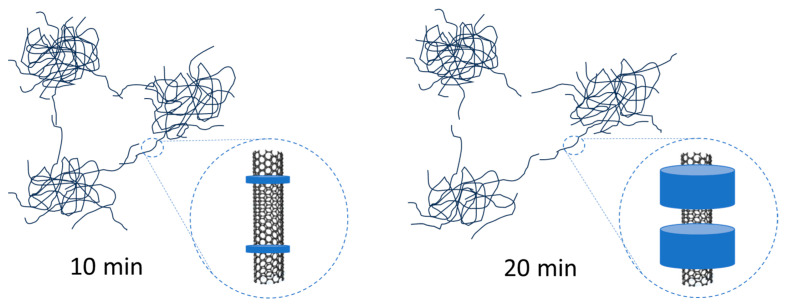
Schematic representation of CNT dispersion within the HDPE matrix with processing times of 10 min and 20 min, with a zoomed image of the CNT surface showing the size of HDPE crystallites.

Nevertheless, the maximum of the melting peak of the nanocomposites produced by the longer melt processing time (i.e., 20 min) is higher than that of the nanocomposites obtained by the shorter melt processing time (i.e., 10 min). This suggests that the crystals of the nanocomposites processed for 20 min are thicker than those of the nanocomposites processed for 10 min. Moreover, from the second heating scan by DSC, we observe that the melting peak shifted to a slightly higher temperature, suggesting the formation of even thicker crystal lamellae. These results are in agreement with what we showed in a previous study on very similar nanocomposites for which we studied their morphology in detail, notably by TEM analysis [32]. In particular, we demonstrated that HDPE crystallites developed during the shear melt-mixing on the CNT surface in the form of a structure known as a Shish-Kebab. This type of structure is well documented in scientific literature [31,32,34]. It can be explained by the fact that CNTs are very good nucleating agents for HDPE crystallization [32,33,34]. We also showed that the longer the shear mixing time in the melt, the larger the HDPE crystallites became. Moreover, we observed that increasing the mixing time from 10 to 20 min did not drastically modify the overall dispersion state of the CNTs in the HDPE matrix, nor did it damage the length of the CNTs (i.e., no significant cut-off CNTs during processing). To better visualize these phenomena, we have schematically represented the morphologies achieved at 10 min and 20 min, respectively, in Figure 2.

Interestingly, the structural differences evidenced by DSC analyses between the nanocomposites do not seem to be sufficient enough to significantly influence the mechanical performances of the produced nanocomposites. Indeed, all the nanocomposites, whatever the processing duration, exhibit very similar tensile and impact behavior (see Table 2). This result can be explained by the fact that mechanical properties are mostly driven by the global rate of crystallinity, which is maintained as quasi-identical for all the nanocomposites regardless of the duration of processing rather than the size of the lamellae.

Conversely, the electrical behaviors of the nanocomposites are strongly affected by their mode of preparation. More precisely, the nanocomposites prepared with the shortest implementation time have a level of electrical resistivity in DC that is significantly lower than those prepared in 20 min.

As mentioned before, CNTs are excellent nucleating agents of HDPE crystallization with HDPE crystal lamellae growth on the surface of CNTs. Moreover, we reported in a previous work based on similar systems [32] that the longer the implementation time, the bigger the size of these lamellae. These crystals of HDPE can push the CNTs away from each other, decreasing the contacts between CNTs as well as blocking the electrons from hopping between CNTs. As a result, an increase in DC electric resistivity is observed with increased processing time in the melt.

To further understand the electrical behavior of these nanocomposite materials, a study of the dielectric properties is carried out.

### 3.2. Dielectric Properties

**Figure 3:**
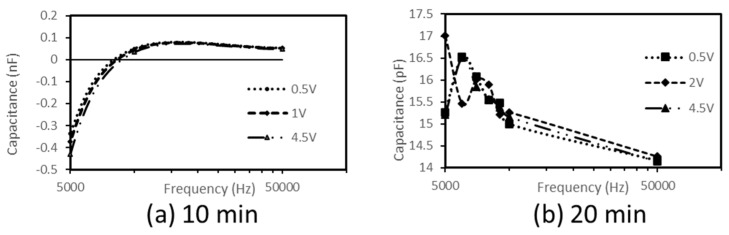
Frequency-dependence of the capacitance of HDPE/4.6%CNT nanocomposites as prepared by melt blending with processing times of (**a**) 10 min and (**b**) 20 min.

**Figure 4:**
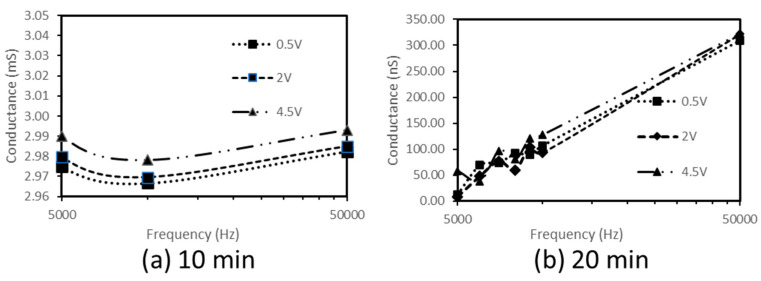
Frequency-dependence of the conductance of HDPE/4.6%CNT nanocomposites as prepared by melt blending with processing times of (**a**) 10 min and (**b**) 20 min.

The frequency dependence of capacitance and conductance were recorded at room temperature on the studied nanocomposites prepared by melt blending for 10 min and 20 min, respectively. The results are presented in Figure 3 and Figure 4.

As observed, the nanocomposite produced by the longer melt processing time, i.e., 20 min, is deprived of any negative capacitance (Figure 3b) and displays an increasing conductance (hence, of non-oscillatory type) (Figure 4b).

On the other hand, remarkably, the nanocomposite melt processed for a shorter processing time, i.e., 10 min, displays a negative capacitance (Figure 3a) and oscillatory conductance (Figure 4a).

The analysis of these results suggests the two following considerations. First, space charge effects leading to current oscillations (conductance follows an oscillatory variation) occur in the crystallite/interface structure obtained with the shorter processing time of 10 min. Second, none of these oscillations exist in the structure characterized by the nanocomposite produced by melt blending for 20 min.

Previous assumptions rely on the work of Doyle [8], who evidenced that negative capacitance in materials with the crystallite/interface structure can take place if space charge effects at the interfaces of crystallites create current oscillations. These oscillations are then supposed to be responsible for NC.

These two crystallite/interface structures produced with processing times of 10 min and 20 min, respectively, only differ from each other in crystallite size. Here, the key factor for these oscillations that lead to NC is the size of the crystallites.

Hence, the technique with a processing time of 10 min produces crystallites with the necessary size to authorize these oscillations, which is not the case with larger crystallites produced with a longer processing time of 20 min.

Both voltage dependence and the increase in NC are typical of systems in which NC relies on the interface [8,13].

The maximum absolute value of NC obtained with the nanocomposite produced by the shorter melt processing time, i.e., 10 min ($\sim 10^{-9}\;F),$ is three orders of magnitude higher than that of poly(3-hexylthiophene) (P3HT) [12] ($\sim 10^{-12}\;F)$ for frequencies lower than 10 KHz for the first case and lower than 0.1 kHz for the second. This result suggests that the HDPE/MWCNT could be utilized for applications requiring very low courant.

The analysis of the frequency trend of the real permittivity (or real part of complex permittivity) of both nanocomposites was also realized.

As observed, the real permittivity of the nanocomposite produced by the shorter processing time (10 min) (Figure 4a) displays positive values almost frequency-independent towards higher frequencies and negative values decreasing towards lower frequencies. This behaviour can be, theoretically, described by the Drude model [38], which gives frequency-dependent real permittivity as follows:

$$\varepsilon_{r}=1-\frac{\omega_{p}^{2}}{\omega^{2}+\omega_{p}^{2}}$$

where $\omega_{p}(\omega_{p}=2\pi f_{p})$ is the plasma frequency, ω is the frequency of electric field, $\omega_{r}$ is the damping parameter, f the electric field frequency, $\varepsilon_{0}$ is the permittivity of vacuum (8.85 × $10^{-12}\;F/m$), $n_{eff}$ is the effective concentration of delocalized electrons, $m_{eff}$ is the effective weight of electron and e is the electron charge (1.6 × $10^{-19}\;C$).

**Figure 5:**
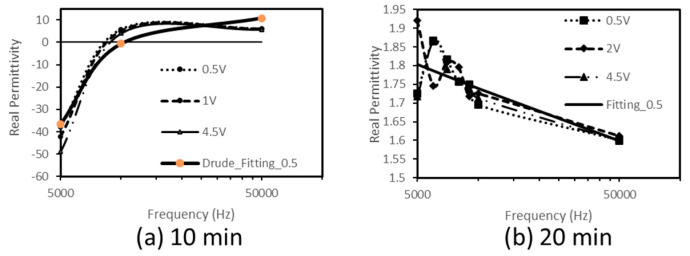
Frequency-dependence trend of the real permittivity of HDPE/4.6%CNT nanocomposites as prepared with melt blending processing times of (**a**) 10 min and (**b**) 20 min.

The curves are almost overlapping (Figure 5a); so, for clarity, we only represent fitting for one case (see full thick line in Figure 5a). The fitted results show that our experimental results present agreement with the Drude model and the fitted plasma frequency of nanocomposite ($f_{p}\sim 10^{4}\;Hz)$ is much lower than that of pure metals ($\sim 10^{15}\;Hz)$ [38]. In this nanocomposite, which behaves as a metal-like material, the negative permittivity can be attributed to the plasma of delocalized electrons implicated in space charge effects at HDPE/MWCNT interfaces, which oscillates for frequencies lower than $f_{p}$.

However, the real permittivity of the nanocomposite produced with a longer processing time (20 min) (Figure 4b) presents exclusively positive values, decreasing with increasing frequencies (for $f>10^{4}Hz$). This variation, which is ascribed to the polarization of polar groups in the sample having more and more difficulty to follow the frequency increase, can be represented by a power law. The experimental results are reasonably fitted with that of a power law (see full thick line, Fitting_0.5 in Figure 5b) suggesting the hopping process of electrons as the main conduction mechanism instead of the oscillating one, as observed in the nanocomposite produced with a shorter processing time (10 min).

## 4. Conclusions

Nanocomposites based on high-density polyethylene (HDPE) matrix and multiwalled carbon nanotubes (CNTs) at a specified percolation threshold (i.e., 4.6 wt.% of CNTs) were successfully prepared by melt processing following two times of mixing (i.e., 10 min being the short time and 20 min being the long time).

This duration change in the process induced microstructural differences in the systems; as a result, very different electrical behaviors were obtained. The nanocomposite issued from the melt-mixing with a shorter time (i.e., 10 min) shows a level of electrical conductivity in direct current mode much higher than the one prepared with a longer time (i.e., 20 min) but was also found to display negative capacitance (NC), whereas the nanocomposite prepared with longer time exhibits none. This switch in behavior could be explained by the size of the HDPE crystallites formed at the surface of CNTs, which became bigger when the processing time lasted longer, leading to the deterioration of the contacts between the CNTs and the destruction of the conductive network by limiting the conductive pathways. This study highlights that it is possible to prepare a material exhibiting NC using an insulating commodity thermoplastic HDPE combined with CNTs at the percolation threshold (i.e., a limited amount of CNTs) following a robust and straightforward melting process under easily controllable conditions allowing the preparation of a nanocomposite with adequate structure. The maximum absolute value found for NC in our study is 1000 times higher than that achieved in semiconducting poly(3-hexylthiophene (P3HT), which is widely implicated in transistor and solar cells for frequencies less than 0.1 kHz under an electric field of 55 V/cm.

This approach, which can be easily upscaled for industrialization and is also more environmentally friendly compared to the solution mixing approach, opens a convenient, alternative way and new opportunities for the development of innovative, lightweight, conformable, smart materials with NC for electronic applications requiring low current.

## Figures and Tables

**Table 1:** Information of sample preparation.

Sample	TMT *	Composition	MFI of the HDPE Matrix
1	10	4.6% CNT + HDPE	8.0 g/10 min at 21.6 kg
2	20	4.6% CNT + HDPE	8.0 g/10 min at 21.6 kg

* TMT: Total mixing time in brabender (min).

**Table 2:** Thermal, mechanical and electrical properties of HDPE/4.6%CNT nanocomposites as prepared by melt blending with processing times of 10 min (sample 1) and 20 min (sample 2).

Sample	T_m_ (°C)	ΔH_m_ (J/g)	DC * (%)	Impact Strength (kJ/m^2^)	Strain at Break (%)	Stress at Break (MPa)	Young Modulus (MPa)	DC-Volume Resistivity (Ω. cm)
1	128	173	60	15 ± 3	171 ± 94	20 ± 3	1495 ± 76	10^4^
2	131	177	61	15 ± 4	171 ± 95	20 ± 4	1495 ± 77	10^14^

* Degree of crystallinity DC = ΔH_m_/293 × 100, where ΔH_m_ is the melting enthalpy of the sample normalized to the amount of HDPE.

## Data Availability

Data will be made available on request.
